# A prospective study on maternal periodontal diseases and neonatal adverse outcomes

**DOI:** 10.2340/aos.v83.40836

**Published:** 2024-06-11

**Authors:** Ping Wen, Huijun Li, Xiaoyi Xu, Feng Zhang, Dan Zhao, Rong Yu, Tianfan Cheng, Hao Wang, Chuanzhong Yang, Wei Qin, Xiuqiao Yang, Jilong Yao, Lijian Jin

**Affiliations:** a Division of Science & Education, Shenzhen Maternity & Child Healthcare Hospital, Shenzhen, China; b Division of Stomatology, Shenzhen Maternity & Child Healthcare Hospital, Shenzhen, China; c Beijing Stomatological Hospital, Capital Medical University, Beijing, China; d Division of Periodontology & Implant Dentistry, Faculty of Dentistry, The University of Hong Kong, Hong Kong SAR, China; e Division of Pediatrics, Shenzhen Maternity & Child Healthcare Hospital, Shenzhen, China; f Division of Neonatology, Shenzhen Maternity & Child Healthcare Hospital, Shenzhen, China; g Division of Obstetrics & Gynecology, Shenzhen Maternity & Child Healthcare Hospital, Shenzhen, China

**Keywords:** Periodontal diseases, probing depth, pregnancy, neonatal adverse outcomes, small-for-gestational age

## Abstract

**Objective:**

It is evident that periodontitis is linked to various adverse pregnancy outcomes. This prospective study explored the potential link of maternal periodontal diseases to neonatal adverse outcomes.

**Materials and Methods:**

A total of 193 generally healthy females in their third trimester (34–36 weeks) of pregnancy were enrolled. All subjects received full-mouth periodontal assessment, and the periodontal inflamed surface area (PISA) was calculated. Demographic data, lifestyles and anthropometric measurements of the neonates (e.g., body length and head circumference) were recorded. Herein, small-for-gestational age (SGA) referred to gender- and age-adjusted birth weight below the 10th percentile in line with the standard reference. Multivariable logistic regression analysis and restricted cubic spline were performed for examining the association of periodontal parameters with SGA.

**Results:**

There were 8.3% (16/193) of neonates with SGA. Significantly positive correlation existed between the percentage of tooth sites with increased probing depth and an elevated risk of SGA (OR: 1.052; *P* < 0.05). Yet, the PISA was positively associated with the risk of SGA (OR: 1.002; *P* < 0.05) as well. No significant link occurred between maternal periodontal status and other neonatal outcome measures.

**Conclusion:**

Within the limitations of this study, the findings suggest that there could be a link between maternal periodontal diseases and neonatal adverse outcomes like SGA. Further investigation is required to clarify the current findings and potential implications for promoting maternal oral/periodontal health and newborn health.

## Background

It is apparent that periodontal (gum) diseases significantly affect oral health and yet have notable associations with COVID-19 and common noncommunicable diseases (NCDs), such as diabetes, cardiovascular disease, inflammatory bowel disease, certain kinds of cancers, rheumatoid arthritis, Alzheimer’s disease and nonalcoholic fatty liver disease [1–7]. The causal connection of periodontitis to its systemic comorbidities is substantiated by emerging scientific evidence on biologically convincible and clinically conceivable mechanisms through which periodontal inflammation could affect the systemic status [8, 9]. As such, periodontitis may aggravate common metabolic–inflammatory diseases and disorders, through upregulating the systemic level of inflammation [4, 8, 10–13].

It is noteworthy that periodontal diseases are frequently present in pregnant women, and the prevalence of maternal periodontitis could go up to 61% [14–16]. The joint consensus statement from the American Academy of Periodontology (AAP) and the European Federation in Periodontology indicates that maternal periodontitis can disturb the healthy condition of fetal-maternal interface, and thereby increase the risk of adverse pregnancy outcomes (APOs) [17]. Up to now, most of the studies in this field address the possible link of periodontal diseases with preterm birth/low birth weight (PTB/LBW), small-for-gestational age (SGA) infants and preeclampsia, as well as the potential effects of periodontal interventions on these disorders [18–28]. Meanwhile, it has been highlighted that periodontal infections and the resultant inflammatory mediators significantly account for the underlying biological mechanisms involved in these disorders [14, 17, 20, 28].

Importantly, SGA neonates are susceptible to various adverse consequences with significant implications for neonatal mortality and morbidity in both developed and developing countries [29]. It has been previously shown that pregnant females with moderate to severe periodontal diseases could exhibit an elevated risk for delivering SGA infants, whereas other possible infections and/or complications during pregnancy that affect fetal growth are not identified and controlled in the study [18]. Moreover, although the recent systematic review and meta-analysis reveal that periodontal diseases markedly increase the risk of PTB and LBW, such notable link of periodontal diseases to SGA could not be identified [30]. Additionally, maternal infection can be one of the possible causes of fetal growth restriction. Taken together, further investigations are needed to clarify these points. As such, this prospective study attempted to elucidate whether there could be a link between maternal periodontal diseases and neonatal adverse outcomes like SGA among generally healthy pregnant women.

## Methods

### Study population

This prospective study consisted of 193 pregnant females (18–35 years at 34–36 gestational weeks), and they were recruited from the Division of Obstetrics & Gynecology at the Shenzhen Maternity & Child Healthcare Hospital (SZMCH). The average follow-up time was 38.0 ± 11.9 days. The participants fulfilled the following criteria: i) singleton pregnancy; ii) full registration with the electronic medical system of SZMCH; iii) normal pre-pregnancy body mass index (BMI); iv) absence of chronic inflammatory diseases; and v) natural pregnancy. The following subjects were excluded: current smokers and those with systemic diseases prior to and/or during pregnancy as well as those receiving periodontal treatments or antibiotics/immunosuppressive agents in the past 6 months (Supplementary Figure 1). Approval for the study was granted by the Ethics Committee from the SZMCH (SFYLS [2020] 013), and this study was conducted according to the guidelines of the Declaration of Helsinki [31]. Prior to this clinical study, informed oral and written consent was given by all the subjects.

### Periodontal assessment

The examination was performed by a calibrated investigator within 1 week of subject recruitment, and the intra-examiner reliability was assessed to be strong having a kappa value of 0.887 [32]. The numbers of tooth loss due to periodontitis and the teeth present excluding 3rd molars were counted. The parameters recorded at six sites per tooth included full-mouth plaque score (FMPS), bleeding on probing (BOP) and probing depth (PD). Moreover, the periodontal inflamed surface area (PISA) was calculated for each subject following an established approach [33].

### Outcome measures of neonates

The primary outcome measure of neonates was small-for-gestational-age (SGA), and it was defined as gender- and age-adjusted birth weight below the 10th percentile following the standard references [34]. Similarly, infants were categorized as large-for-gestational-age (LGA) or appropriate for their gestational age (AGA) respectively, when the gender- and age-adjusted birth weight was above 90th percentile or between the 10th and 90th percentile [34]. The essential information (e.g., gender, gestational age and birth weight) was extracted from the electronic medical system of SZMCH, and well matched to their mothers. Head circumference (cm), birth length (cm) and Apgar score in 5 min were collected from the electronic system as well.

### Statistical analysis

All subjects were categorized into SGA, AGA and LGA groups according to the outcome measures of their neonates. Continuous variables were shown as mean ± SD or median (interquartile ranges) appropriately, while categorical ones were presented with counts and percentages. Mann-Whitney U test examined the differences in periodontal parameters (continuous variables) between SGA and AGA groups or LGA and AGA groups. Whereas Chi-square test was undertaken to assess the inter-group differences in socio-demographic characteristics.

To further explore the association of periodontal parameters with the outcome measures of neonates, logistic regression models were designed for facilitating the analysis. Various potential confounders in the multivariable models were adjusted (i.e., maternal age, educational attainment, household monthly income, pre-pregnancy BMI and pregnancy history). The interconnections of maternal age with periodontal parameters were tested as well. Given an a priori hypothesis that one percentage of sites with increased BOP% or PD ≥ 4 mm might not evenly reflect the exacerbation of periodontal conditions, the restricted cubic spline (RCS) were employed to further examine the potential non-linear associations of periodontal parameters with the incidence of SGA or LGA on the basis of the logistic regression models. The 5^th^, 50^th^ and 95^th^ percentiles were kept as the knots to set the median of the total population as the reference. The link of maternal periodontal parameters with neonatal body length and head circumference was assessed as well, by using the models established. Statistical analyses and curve fitting were undertaken with the SAS 9.4 software and R (version 4.0), respectively. A two-side *P*-value < 0.05 was determined to be statistically significant.

## Results

### Demographics/periodontal conditions of the pregnant females and the health conditions of their neonates

The demographic characteristics and periodontal status of all 193 pregnant females are presented in Table 1. Overall, 51.8% of subjects were 30 years old or below. Majority of them obtained university or higher degrees (74.6%), and 47.7% earned over 20,000 CNY household income per month. A total of 50.8% had never been pregnant, and 29.1% had over one miscarriage experience. Regarding periodontal parameters, the median (IQR) of FMPS, BOP% and sites% with PD ≥ 4 mm was 80.0 (65.0, 93.0), 20.8 (9.5, 34.7), and 4.0 (0.0, 11.0), respectively. Only 23 subjects exhibited PD ≥ 6 mm. The median (IQR) of PISA was 244.4 (99.8, 517.3) mm^2^. Most participants had over 28 teeth and none lost tooth due to periodontitis (data not shown). Overall, the median (IQR) of gestational age at delivery was 39.0 (39.0, 40.0) weeks. Four infants were born prematurely. The mean of birth weight was 3,289.5 ± 419.2 g. Of all the newborns, two were born below 2,500 g, and 10 were born over 4,000 g. Moreover, the median (IQR) of body length and head circumference was 50.0 (50.0, 50.0) cm and 34.0 (34.0, 35.0) cm, respectively. Moreover, the median (IQR) of Apgar score at 5 min was 10.0 (10.0, 10.0). No birth defects were reported.

**Table 1:** Demographic characteristics, periodontal parameters and outcome measures of neonates in 193 pregnant females.

Characteristics	Total (*N* = 193)	AGA (*n* = 155)	SGA (*n* = 16)	LGA (*n* = 22)
***Sociodemographic characteristics***				
**Age, years^a^**				
≤ 30	100 (51.8)	77 (49.7)	13 (81.3)	10 (45.5)
> 30	93 (48.2)	78 (50.3)	3 (18.8)	12 (54.6)
**Education level^b^**				
High school or lower	49 (25.4)	33 (21.3)	5 (31.3)	11 (50.0)
University level or higher	144 (74.6)	122 (78.7)	11 (68.8)	11 (50.0)
**Household monthly income (CNY)**				
< 9,000	42 (21.8)	36 (23.2)	1 (6.3)	5 (22.7)
9,000–19,999	59 (30.6)	45 (29.0)	8 (50.0)	6 (27.3)
≥ 20,000	92 (47.7)	74 (47.7)	7 (43.8)	11 (50.0)
**Gestational history**				
No	98 (50.8)	79 (51.0)	11 (68.8)	7 (31.8)
Yes	95 (49.2)	76 (49.0)	5 (31.3)	15 (68.2)
***Periodontal parameters***				
**FMPS**	80.0 (65.0, 93.0)	80.0 (65.0, 93.0)	79.5 (62.5, 98.0)	81.5 (68.0, 91.0)
**BOP, %**	20.8 (9.5, 34.7)	19.3 (9.5, 33.3)	23.4 (8.6, 49.7)	25.1 (12.3, 38.1)
**PD** ≥ **4 mm, sites%**	4.0 (0.0, 11.0)	3.0 (0.0, 10.0)	4.0 (0.0, 27.5)	5.0 (0.0, 19.0)
**PISA**	244.4 (99.8, 517.3)	239.4 (94.9, 451.3)	288.0 (92.1, 776.3)	269.6 (160.0, 590.5)
***Outcome measures of neonates***				
**Delivery week**	39.0 (39.0, 40.0)	39.0 (38.0, 40.0)	39.0 (39.0, 40.0)	39.0 (38.0, 40.0)
**Gender**				
Male	105 (54.4)	89 (57.4)	6 (37.5)	10 (45.5)
Female	88 (45.6)	66 (42.6)	10 (62.5)	12 (54.6)
**Birth weight, g^a, b^**	3289.5 ± 419.2	3244.6 ± 305.9	2706.25 ± 186.5	4029.5 ± 218.5
**Birth length, cm^a, b^**	50.0 (50.0, 50.0)	50.0 (50.0, 50.0)	49.0 (48.0, 49.0)	51.0 (51.0, 52.0)
**Head circumference, cm ^a, b^**	34.0 (34.0, 35.0)	34.0 (34.0, 34.0)	33.0 (33.0, 34.0)	35.0 (35.0, 36.0)
**APGAR 5 min score**	10.0 (10.0, 10.0)	10.0 (10.0, 10.0)	10.0 (10.0, 10.0)	10.0 (10.0, 10.0)

a *P*-value < 0.05 for t-test or Chi-square test as appropriate comparing SGA group with AGA group for variables.

b *P*-value < 0.05 for t-test or Chi-square test as appropriate comparing LGA group with AGA group for variables.

Notably, 8.3% (16/193) of the newborns were identified as SGA, while 11.4% (22/193) exhibited LGA. Except for pregnant history, other characteristics of the mothers were comparable between the groups (SGA vs. AGA and LGA vs. AGA). Whereas, significant differences occurred in birth weight, birth length and head circumference in the groups with different neonatal weights (Table 1).

### Periodontal status of pregnant females among the SGA, LGA and AGA groups

The median (IQR) of FMPS, BOP% and sites% with PD ≥ 4 mm and PISA in SGA group was 79.5 (62.5, 98.0), 23.4 (8.6, 49.7), 4.0 (0.0, 27.5) and 288.0 (92.1, 1776.3) mm^2^, respectively; and those in LGA were 81.5 (68.0, 91.0), 25.1 (12.3, 38.1), 5.0 (0.0, 19.0) and 269.6 (160.0, 590.5) mm^2^, respectively. While the median of these parameters in AGA was 80.0 (65.0, 93.0), 19.3 (9.5, 33.3), 3.0 (0.0, 10.0) and 239.4 (94.9, 451.3) mm^2^, respectively. Comparing with AGA group, both SGA and LGA groups showed no significant difference in the periodontal parameters (Table 1 and Supplementary Figure 2).

### Multivariable logistic regression modelling of maternal periodontal parameters to the neonates’ outcome measures

The multivariable logistic regression models revealed that both maternal PD ≥ 4 mm (sites%) and PISA were positively correlated with the presence of SGA (*P* < 0.05) (Table 2). Notably, the subjects with a greater number of tooth sites with PD ≥ 4 mm and increased PISA exhibited risk ratios of 1.048 (95%CI:1.008, 1.089; *P* < 0.05) and 1.001 (95%CI:1.000, 1.003; *P* < 0.05), respectively for SGA after adjusting for maternal age (Model 1), with reference to the counterparts. Such positive correlations remained to be significant for PD ≥ 4 mm, after further adjusting for potential confounders including educational attainment, household monthly income, pre-pregnancy BMI and pregnancy history (Models 2 & 3) as well as for PISA after adjusting all these variables (Model 3). Whereas, BOP% was not significantly correlated with SGA. No significant interaction existed between age and periodontal parameters accounting for SGA. There was no significant association of periodontal parameters with the prevalence of LGA, with reference to the AGA neonates.

**Table 2:** The association of periodontal parameters in pregnant females with their neonates’ presence of SGA or LGA examined via multivariable logistic regression models.

Periodontal parameters	SGA vs. AGA (*n* = 171)						
	Model 1	*P*-value	Model 2	*P*-value	Model 3	*P-*value	*P* for interaction
**BOP, %**	1.025 (0.997, 1.054)	0.078	1.025 (0.995, 1.057)	0.104	1.028 (0.996, 1.061)	0.085	0.120
**PD ≥ 4 mm, %**	1.048 (1.008, 1.089)	**0.016**	1.050 (1.006, 1.096)	**0.023**	1.052 (1.008, 1.100)	**0.020**	0.206
**PISA**	1.001 (1.000, 1.003)	**0.038**	1.001 (1.000, 1.003)	**0.053**	1.002 (1.000, 1.003)	**0.041**	0.170
Periodontal parameters	LGA vs. AGA (*n* = 177)						
	Model 1	*P*-value	Model 2	*P*-value	Model 3	*P-*value	*P* for interaction
**BOP, %**	1.017 (0.989, 1.044)	0.225	1.012 (0.984, 1.041)	0.383	1.010 (0.981, 1.039)	0.495	0.988
**PD ≥ 4 mm, %**	1.031 (0.994, 1.066)	0.082	1.024 (0.986, 1.062)	0.209	1.025 (0.987, 1.066)	0.192	0.618
**PISA**	1.001 (1.000, 1.002)	0.114	1.001 (0.999, 1.002)	0.289	1.001 (0.999, 1.002)	0.309	0.490

Model 1 adjusted for maternal age.

Model 2 adjusted for maternal age, education level and household monthly income.

Model 3 adjusted for all variables in model 2 with additional added pre-pregnancy BMI and pregnancy history.

**Figure 1:**
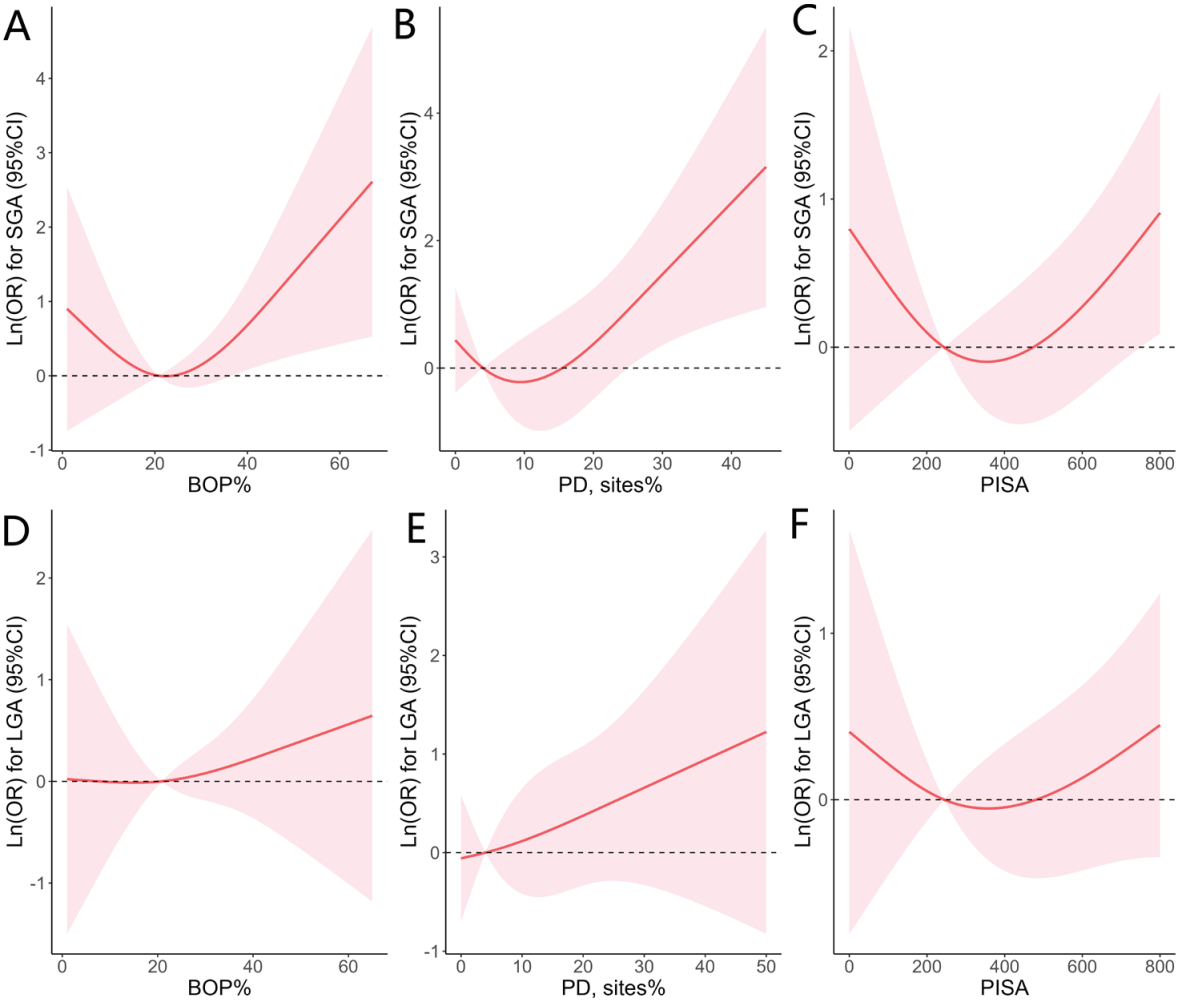
Non-linear associations of maternal periodontal parameters in pregnant females with the risk of delivering SGA and LGA neonates. The y-axis is a log scale of odds ratio (OR) in which the red solid line delineates multivariate-adjusted OR and the dashed lines show the 95% CIs. A knot is located at the 5th, 50th, and 95th percentiles for BOP% (**A** and **D**), PD sites% (**B** and **E**) or PISA (**C** and **F)**. All models were adjusted for maternal age, education level, household monthly income, pre-pregnancy BMI, and pregnancy history. BOP: bleeding on probing; PD: probing depth; BMI: body mass index; PISA: the periodontal inflamed surface area.

The RCS was used to further curve the models and visualize the correlation of periodontal parameters to the delivery of SGA or LGA neonates (Figure 1). A marginally positive association existed between BOP% and risk of SGA (*P* value for non-linearity = 0.055, Figure 1A). There was no detectable non-linear relationship between PD ≥ 4 mm (sites%) and SGA (Figure 1B) or between PISA and SGA (Figure 1C). Likewise, there was no non-linear correlation of these two parameters with the risk of delivering LGA newborns (Figures 1D–F). Furthermore, the associations of periodontal parameters with the quantitative measures of newborns (weight, length and head circumference) were also assessed using linear regression models. There was no significant correlation of BOP, PD ≥ 4 mm and PISA with these neonatal outcome measures (Table 3).

**Table 3:** The association of periodontal parameters in pregnant females with their neonates’ outcome measures via linear regression models.

Periodontal status	*β*	S.E.	*t*	*P*-value
***Birth weight, g***				
BOP, %	−0.061	1.766	−0.030	0.973
PD ≥ 4 mm, sites%	1.180	2.56	0.460	0.645
PISA	6.513 × 10^-3^	9.048 × 10^-2^	0.072	0.943
***Birth length, cm***				
BOP, %	−0.006	0.004	−1.400	0.163
PD ≥ 4 mm, sites%	−0.005	0.006	−0.740	0.463
PISA	−0.0003	0.0002	−1.343	0.181
***Head circumference, cm***				
BOP, %	0.0004	0.003	0.130	0.898
PD ≥ 4 mm, sites%	0.003	0.005	0.670	0.504
PISA	2.220 × 10^-5^	1.719 × 10^-4^	0.129	0.897

## Discussion

This prospective study evaluated the potential association of maternal periodontal status with neonatal adverse outcomes in a cohort of females from a metropolitan city in South China, and elaborated on the potential clinical implications. The overall findings suggest that existing maternal periodontal condition (e.g., presence of multiple sites with BOP, PD ≥ 4 mm and increased PISA) may possibly account for fetal growth restriction, and hence increase the likelihood of delivering SGA infants.

Pregnant females are basically susceptible to detectable changes in oral cavity, owing to altered lifestyles and profiles of progesterone and estrogen levels during pregnancy. Subsequently, the marked increase in sex hormones crucially alters the condition of periodontal tissues with amplified inflammatory responses. Such resultant change could, to some extent, occur even with reasonable oral hygiene practice [35]. Notably, pregnancy accounts for an increased level of periodontal inflammation, along with accumulated burden of dysbiotic biofilms and the resultant dysregulated host responses; consequently, it could aggravate untreated existing periodontal diseases and the related systemic inflammatory comorbidities [36–39]. Conceivably, the risk of gingivitis and periodontitis can elevate during pregnancy period [40], particularly in susceptible individuals.

The link of oral/periodontal health with various APOs has been well documented [24, 41–43], since the first paper in this theme was published in 1996 [25]. Indeed, a recent cohort study demonstrates that the severity of periodontitis identified following the current new classification links to various APOs and adverse neonatal outcomes [21]. Currently, two underlying pathogenic mechanisms have been proposed for periodontal diseases-triggered APOs, namely direct translocation of dysbiotic oral microbiomes and/or their virulent components to the fetal-placenta unit or indirectly mouth-derived inflammatory mediators affecting the unit [9, 20]. In the latter pathway, periodontal niches-derived inflammatory mediators significantly enhance the expression and production of pro-inflammatory cytokines and prostaglandins in the fetal-placenta unit via the general circulation, thereby resulting in uterine contraction and rupture of fetal membranes [20].

With regard to the birth of SGA infants, maternal health conditions are of great importance for fetal growth. Several studies have revealed that the delivery of SGA neonates may be attributed to maternal pre-pregnancy underweight and some socioeconomic factors like poor education level [44, 45]. The underlying mechanism of SGA may be related to placenta dysfunction and dyslipidemia [46, 47]. Furthermore, it has been shown that the risk of SGA infant delivery may increase with the upregulation of maternal inflammatory biomarkers in periodontitis patients, suggesting that both local and systemic inflammation would account for the delivery of SGA infants [18]. Notably, this study demonstrates that increased probing depth is significantly associated with the delivery of SGA infants, in agreement with the previous findings [18]. In addition, it has also been documented that periodontal status is significantly related to fetal growth parameters, suggesting the fact that existing periodontal inflammation contributes to fetal growth restriction [48, 49]. As such, in addition to the commonly used periodontal parameters, the emerging variable so called PISA has been measured for quantifying the burden of periodontal inflammation in the present study [32, 33]. Interestingly, the PISA shows a significantly positive correlation with the risk of SGA. Collectively, these findings reveal that maternal periodontal diseases could potentially increase the risk of neonatal adverse outcomes like SGA. Further longitudinal studies are required to clarify the current findings and elaborate the clinical implications.

Oral care is an essential component of comprehensive maternity healthcare. It is noteworthy that the AAP has published a practical guideline on periodontal healthcare of pregnant women, for promoting maternal health and the well-being of newborns [50]. Currently, the prevalence of maternal periodontal diseases remains to be relatively high in both developed and developing countries [51, 52]. Yet, the public is not fully aware of oral/periodontal health, and the mothers-to-be often could not get access to adequate oral care on a regular basis [52]. Our recent work on the considerable link between maternal periodontal diseases and systemic disorders further highlights the profound importance of integrating oral healthcare into professional maternal schemes for the optimal health and well-being of pregnant females and their newborn babies [32, 53]. Since periodontal diseases are a modifiable risk factor of neonatal adverse outcomes, it is strongly suggested that both oral healthcare professionals and obstetricians should raise their awareness of periodontal health in child-bearing women. Meanwhile, appropriate preventive measures and in-time periodontal care should be undertaken, along with proactive health education and awareness on upholding healthy lifestyles in the public community. Herein, our recent work on the proposed pregnancy-integrated FDI scoring system could facilitate convenient screening of maternal periodontal diseases in daily practice [54] for promoting oral/periodontal health of mothers-to-be and reducing the risk of adverse outcomes of their neonates.

There are certain limitations for this study. Firstly, the exact linking profiles of periodontal diseases and neonatal adverse outcomes may not be fully reflected in the current cohort of pregnant women with relatively young age, self-reported healthy condition and low proportion of subjects with PD ≥ 6 mm. Secondly, a more representative and large-sample cohort would be preferred via random selection from various hospitals in the area for enriching subject diversities and addressing notable variables (e.g., socioeconomic status, educational attainment and access to oral/periodontal healthcare). Thirdly, as oral radiographic examination was not commonly preferred by the participants during pregnancy and past dental records were not available for most of them, yet there was relatively low prevalence of severe periodontitis in the cohort, the subjects were not categorized with the current AAP/EFP classification. Further follow-up investigations are required to elaborate the translational values in clinical practice. Meanwhile, the pathogenic pathways accounting for the underlying linkage of maternal periodontal diseases with neonatal adverse outcomes need to be explored and identified, favorably via the combined approaches of metagenomics, metabolomics and proteomics.

## Conclusions

Within the notable limitations of the present investigation, the findings suggest that there could be a link between maternal periodontal diseases and the delivery of SGA infants. As such, proactive pre-pregnancy oral evaluation and professional healthcare need to be undertaken for children-bearing women to enhance their maternal wellbeing and newborn health.

## Supplementary Material



## Data Availability

The data that support the findings of this study are available from the corresponding author upon reasonable request.

## References

[1] Genco RJ, Sanz M. Clinical and public health implications of periodontal and systemic diseases: an overview. Periodontol 2000. 2020;83(1):7–13. 10.1111/prd.12344 32385880

[2] Potempa J, Mydel P, Koziel J. The case for periodontitis in the pathogenesis of rheumatoid arthritis. Nat Rev Rheumatol. 2017;13(10):606–20. 10.1038/nrrheum.2017.132 28835673

[3] Acharya C, Sahingur SE, Bajaj JS. Microbiota, cirrhosis, and the emerging oral-gut-liver axis. JCI Insight. 2017;2(19):e94416. 10.1172/jci.insight.94416 28978799 PMC5841881

[4] Schenkein HA, Papapanou PN, Genco R, Sanz M. Mechanisms underlying the association between periodontitis and atherosclerotic disease. Periodontol 2000. 2020;83(1):90–106. 10.1111/prd.12304 32385879

[5] Anand PS, Jadhav P, Kamath KP, Kumar SR, Vijayalaxmi S, Anil S. A case-control study on the association between periodontitis and coronavirus disease (COVID-19). J Periodontol. 2022;93(4):584–90. 10.1002/JPER.21-0272 34347879

[6] Wang Y, Deng H, Pan Y, Jin L, Hu R, Lu Y, et al. Periodontal disease increases the host susceptibility to COVID-19 and its severity: a Mendelian randomization study. J Transl Med. 2021;19(1):528. 10.1186/s12967-021-03198-2 34952598 PMC8708510

[7] Marouf N, Cai W, Said KN, Daas H, Diab H, Chinta VR, et al. Association between periodontitis and severity of COVID-19 infection: a case-control study. J Clin Periodontol. 2021;48(4):483–91. 10.1111/jcpe.13435 33527378 PMC8014679

[8] Hajishengallis G, Chavakis T. Local and systemic mechanisms linking periodontal disease and inflammatory comorbidities. Nat Rev Immunol. 2021;21(7):426–40. 10.1038/s41577-020-00488-6 33510490 PMC7841384

[9] Hajishengallis G. Periodontitis: from microbial immune subversion to systemic inflammation. Nat Rev Immunol. 2015;15(1):30–44. 10.1038/nri3785 25534621 PMC4276050

[10] D’Aiuto F, Gkranias N, Bhowruth D, Khan T, Orlandi M, Suvan J, et al. Systemic effects of periodontitis treatment in patients with type 2 diabetes: a 12 month, single-centre, investigator-masked, randomised trial. Lancet Diabetes Endocrinol. 2018;6(12):954–65. 10.1016/S2213-8587(18)30038-X 30472992

[11] Bajaj JS, Matin P, White MB, Fagan A, Deeb JG, Acharya C, et al. Periodontal therapy favorably modulates the oral-gut-hepatic axis in cirrhosis. Am J Physiol Gastrointest Liver Physiol. 2018;315(5):G824–37. 10.1152/ajpgi.00230.2018 30118351 PMC6293251

[12] Genco RJ, Van Dyke TE. Prevention: reducing the risk of CVD in patients with periodontitis. Nat Rev Cardiol. 2010;7(9):479–80. 10.1038/nrcardio.2010.120 20725103

[13] Tonetti MS. Periodontitis and risk for atherosclerosis: an update on intervention trials. J Clin Periodontol. 2009;36(Suppl 10):15–19. 10.1111/j.1600-051X.2009.01417.x 19432627

[14] Raju K, Berens L. Periodontology and pregnancy: An overview of biomedical and epidemiological evidence. Periodontol 2000. 2021;87(1):132–42. 10.1111/prd.12394 34463990

[15] Usin MM, Tabares SM, Parodi RJ, Sembaj A. Periodontal conditions during the pregnancy associated with periodontal pathogens. J Investig Clin Dent. 2013;4(1):54–9. 10.1111/j.2041-1626.2012.00137.x 23335585

[16] Manau C, Echeverria A, Agueda A, Guerrero A, Echeverria JJ. Periodontal disease definition may determine the association between periodontitis and pregnancy outcomes. J Clin Periodontol. 2008;35(5):385–97. 10.1111/j.1600-051X.2008.01222.x 18341599

[17] Sanz M, Kornman K, working group 3 of the joint EFPAAP workshop. Periodontitis and adverse pregnancy outcomes: consensus report of the Joint EFP/AAP Workshop on Periodontitis and Systemic Diseases. J Periodontol. 2013;84(4 Suppl):S164–9. 10.1902/jop.2013.1340016 23631576

[18] Boggess KA, Beck JD, Murtha AP, Moss K, Offenbacher S. Maternal periodontal disease in early pregnancy and risk for a small-for-gestational-age infant. Am J Obstet Gynecol. 2006;194(5):1316–22. 10.1016/j.ajog.2005.11.059 16647916

[19] Chambrone L, Guglielmetti MR, Pannuti CM, Chambrone LA. Evidence grade associating periodontitis to preterm birth and/or low birth weight: I. A systematic review of prospective cohort studies. J Clin Periodontol. 2011;38(9):795–808. 10.1111/j.1600-051X.2011.01755.x 21707694

[20] Figuero E, Han YW, Furuichi Y. Periodontal diseases and adverse pregnancy outcomes: mechanisms. Periodontol 2000. 2020;83(1):175–88. 10.1111/prd.12295 32385886

[21] Heo JS, Ahn KH, Park JS. Radiological screening of maternal periodontitis for predicting adverse pregnancy and neonatal outcomes. Sci Rep. 2020;10(1):21266. 10.1038/s41598-020-78385-0 33277556 PMC7718227

[22] Ide M, Papapanou PN. Epidemiology of association between maternal periodontal disease and adverse pregnancy outcomes – systematic review. J Periodontol. 2013;84(4 Suppl):S181–94. 23631578 10.1902/jop.2013.134009

[23] Iheozor-Ejiofor Z, Middleton P, Esposito M, Glenny AM. Treating periodontal disease for preventing adverse birth outcomes in pregnant women. Cochrane Database Syst Rev. 2017;6:CD005297. 10.1002/14651858.CD005297.pub3 28605006 PMC6481493

[24] Marquez-Corona ML, Tellez-Giron-Valdez A, Pontigo-Loyola AP, Islas-Zarazua R, Robles-Bermeo NL, Gonzalez-Lopez BS, Medina-Solis CE. Preterm birth associated with periodontal and dental indicators: a pilot case-control study in a developing country. J Matern Fetal Neonatal Med. 2021;34(5):690–5. 31035800 10.1080/14767058.2019.1613363

[25] Offenbacher S, Katz V, Fertik G, Collins J, Boyd D, Maynor G, et al. Periodontal infection as a possible risk factor for preterm low birth weight. J Periodontol. 1996;67(10 Suppl):1103–13. 10.1902/jop.1996.67.10.1103 8910829

[26] Jung E, Romero R, Yeo L, Gomez-Lopez N, Chaemsaithong P, Jaovisidha A, et al. The etiology of preeclampsia. Am J Obstet Gynecol. 2022;226(2S):S844–66. 35177222 10.1016/j.ajog.2021.11.1356 PMC8988238

[27] Spivakovsky S. Periodontal treatment for the prevention of adverse birth outcomes. Evid Based Dent. 2018;19(1):12–13. 10.1038/sj.ebd.6401286 29568020

[28] Komine-Aizawa S, Aizawa S, Hayakawa S. Periodontal diseases and adverse pregnancy outcomes. J Obstet Gynaecol Res. 2019;45(1):5–12. 30094895 10.1111/jog.13782

[29] Rijken MJ, De Livera AM, Lee SJ, Boel ME, Rungwilailaekhiri S, Wiladphaingern J, et al. Quantifying low birth weight, preterm birth and small-for-gestational-age effects of malaria in pregnancy: a population cohort study. PLoS One. 2014;9(7):e100247. 10.1371/journal.pone.0100247 24983755 PMC4077658

[30] Zhang Y, Feng W, Li J, Cui L, Chen ZJ. Periodontal disease and adverse neonatal outcomes: a systematic review and meta-analysis. Front Pediatr. 2022;10:799740. 10.3389/fped.2022.799740 35601423 PMC9114501

[31] World Medical Association. World Medical Association Declaration of Helsinki: ethical principles for medical research involving human subjects. JAMA. 2013;310(20):2191–4. 24141714 10.1001/jama.2013.281053

[32] Zhao D, Cheng T, Hu D, Xu X, Zhang F, Yu R, et al. Maternal periodontal diseases affect the leukocyte profiles of umbilical cord blood: A cohort study. Oral Dis. 2024;30:2533–45. 10.1111/odi.14683 37485723

[33] Miki K, Kitamura M, Hatta K, Kamide K, Gondo Y, Yamashita M, et al. Periodontal inflamed surface area is associated with hs-CRP in septuagenarian Japanese adults in cross-sectional findings from the SONIC study. Sci Rep. 2021;11(1):14436. 34262126 10.1038/s41598-021-93872-8 PMC8280099

[34] Huang XY, Liu HL, Lei M, Mai HF, Lian CH, Li YC. Intrauterine growth curves for body weight, body length, head circumference, chest circumference, and crown-rump length in 16 887 neonates with a gestational age of 27–42 weeks in Shenzhen, China. Zhongguo Dang Dai Er Ke Za Zhi. 2017;19(8):877–86. [Chinese] 28774362 10.7499/j.issn.1008-8830.2017.08.007 PMC7390042

[35] Wu M, Chen SW, Su WL, Zhu HY, Ouyang SY, Cao YT, et al. Sex hormones enhance gingival inflammation without affecting IL-1beta and TNF-alpha in periodontally healthy women during pregnancy. Mediators Inflamm. 2016;2016:4897890. 27034591 10.1155/2016/4897890 PMC4791509

[36] Kumar PS. Sex and the subgingival microbiome: do female sex steroids affect periodontal bacteria? Periodontol 2000. 2013;61(1):103–24. 10.1111/j.1600-0757.2011.00398.x 23240946

[37] Nuriel-Ohayon M, Neuman H, Koren O. Microbial changes during pregnancy, birth, and infancy. Front Microbiol. 2016;7:1031. 27471494 10.3389/fmicb.2016.01031 PMC4943946

[38] Fujiwara N, Tsuruda K, Iwamoto Y, Kato F, Odaki T, Yamane N, et al. Significant increase of oral bacteria in the early pregnancy period in Japanese women. J Investig Clin Dent. 2017;8(1):e12189. 10.1111/jicd.12189 26345599

[39] Balan P, Chong YS, Umashankar S, Swarup S, Loke WM, Lopez V, et al. Keystone species in pregnancy gingivitis: a snapshot of oral microbiome during pregnancy and postpartum period. Front Microbiol. 2018;9:2360. 10.3389/fmicb.2018.02360 30356676 PMC6189292

[40] Mascarenhas P, Gapski R, Al-Shammari K, Wang HL. Influence of sex hormones on the periodontium. J Clin Periodontol. 2003;30(8): 671–81. 10.1034/j.1600-051X.2003.00055.x 12887335

[41] Bobetsis YA, Graziani F, Gursoy M, Madianos PN. Periodontal disease and adverse pregnancy outcomes. Periodontol 2000. 2020;83(1):154–74. 32385871 10.1111/prd.12294

[42] Pockpa ZAD, Soueidan A, Koffi-Coulibaly NT, Limam A, Badran Z, Struillou X. Periodontal diseases and adverse pregnancy outcomes: review of two decades of clinical research. Oral Health Prev Dent. 2021;19(1):77–83. 33491381 10.3290/j.ohpd.b898969 PMC11641288

[43] Xu B, Han YW. Oral bacteria, oral health, and adverse pregnancy outcomes. Periodontol 2000. 2022;89(1):181–9. 35244963 10.1111/prd.12436

[44] Lefizelier E, Misbert E, Brooks M, Thuaut AL, Winer N, Ducarme G. Preterm birth and small-for-gestational age neonates among prepregnancy underweight women: a case-controlled study. J Clin Med. 2021;10(24) 10.3390/jcm10245733 PMC8709329 34945028

[45] Ruiz M, Goldblatt P, Morrison J, Kukla L, Švancara J, Riitta-Järvelin M, et al. Mother’s education and the risk of preterm and small for gestational age birth: a DRIVERS meta-analysis of 12 European cohorts. J Epidemiol Community Health. 2015;69(9):826–33. 10.1136/jech-2014-205387 25911693 PMC4552914

[46] Kim SY, Lee SM, Kwon GE, Kim BJ, Koo JN, Oh IH, et al. Maternal dyslipidemia and altered cholesterol metabolism in early pregnancy as a risk factor for small for gestational age neonates. Sci Rep. 2021;11(1):21066. 34702839 10.1038/s41598-021-00270-1 PMC8548295

[47] Parra-Saavedra M, Crovetto F, Triunfo S, Savchev S, Peguero A, Nadal A, et al. Placental findings in late-onset SGA births without Doppler signs of placental insufficiency. Placenta. 2013;34(12):1136–41. 10.1016/j.placenta.2013.09.018 24138874

[48] Lafaurie GI, Gómez LA, Montenegro DA, De Avila J, Tamayo MC, Lancheros MC, et al. Periodontal condition is associated with adverse perinatal outcomes and premature rupture of membranes in low-income pregnant women in Bogota, Colombia: a case-control study. J Matern Fetal Neonatal Med. 2020;33(1):16–23. 29852806 10.1080/14767058.2018.1484092

[49] Takeuchi N, Ekuni D, Irie K, Furuta M, Tomofuji T, Morita M, Watanabe T. Relationship between periodontal inflammation and fetal growth in pregnant women: a cross-sectional study. Arch Gynecol Obstet. 2013;287(5):951–7. 10.1007/s00404-012-2660-4 23224698

[50] American Academy of Periodontology. American Academy of Periodontology statement regarding periodontal management of the pregnant patient. J Periodontol. 2004;75(3):495. 10.1902/jop.2004.75.3.495 15088891

[51] Jiao J, Jing W, Si Y, Feng X, Tai B, Hu D, et al. The prevalence and severity of periodontal disease in Mainland China: data from the Fourth National Oral Health Survey (2015–2016). J Clin Periodontol. 2021;48(2):168–79. 33103285 10.1111/jcpe.13396

[52] Erchick DJ, Rai B, Agrawal NK, Khatry SK, Katz J, LeClerq SC, et al. Oral hygiene, prevalence of gingivitis, and associated risk factors among pregnant women in Sarlahi District, Nepal. BMC Oral Health. 2019;19(1):2. 10.1186/s12903-018-0681-5 30611255 PMC6321675

[53] Zhang F, Zhao D, Xu X, Wen P, Li H, Yu R, et al. Periodontitis links to concurrent metabolic disorders and abnormal liver function in pregnant women. Oral Dis. 2024; 30(2):697-709. 10.1111/odi.14364 36039534

[54] Li HJ, Zhao D, Xu X, Yu R, Zhang F, Cheng T, et al. Diagnostic performance of the AAP/EFP classification and the CDC/AAP case definition among pregnant women and a practical screening tool for maternal periodontal diseases. J Periodontal Res. 2022;57(5):960–8. 10.1111/jre.13032 35815371 PMC9543595

