# Supplementary material for: A prospective study on maternal periodontal diseases and neonatal adverse outcomes
Source: Acta Odontol Scand. 2024 Jun 11;83:40836. doi: 10.2340/aos.v83.40836 (PMC11302652; doi:10.2340/aos.v83.40836)
Supplement: Supplementary file 1 [file AOS-83-40836-s1.pdf]

Supplementary material has been published as submitted. It has not been copyedited or typeset by Acta Odontologica Scandinavica.

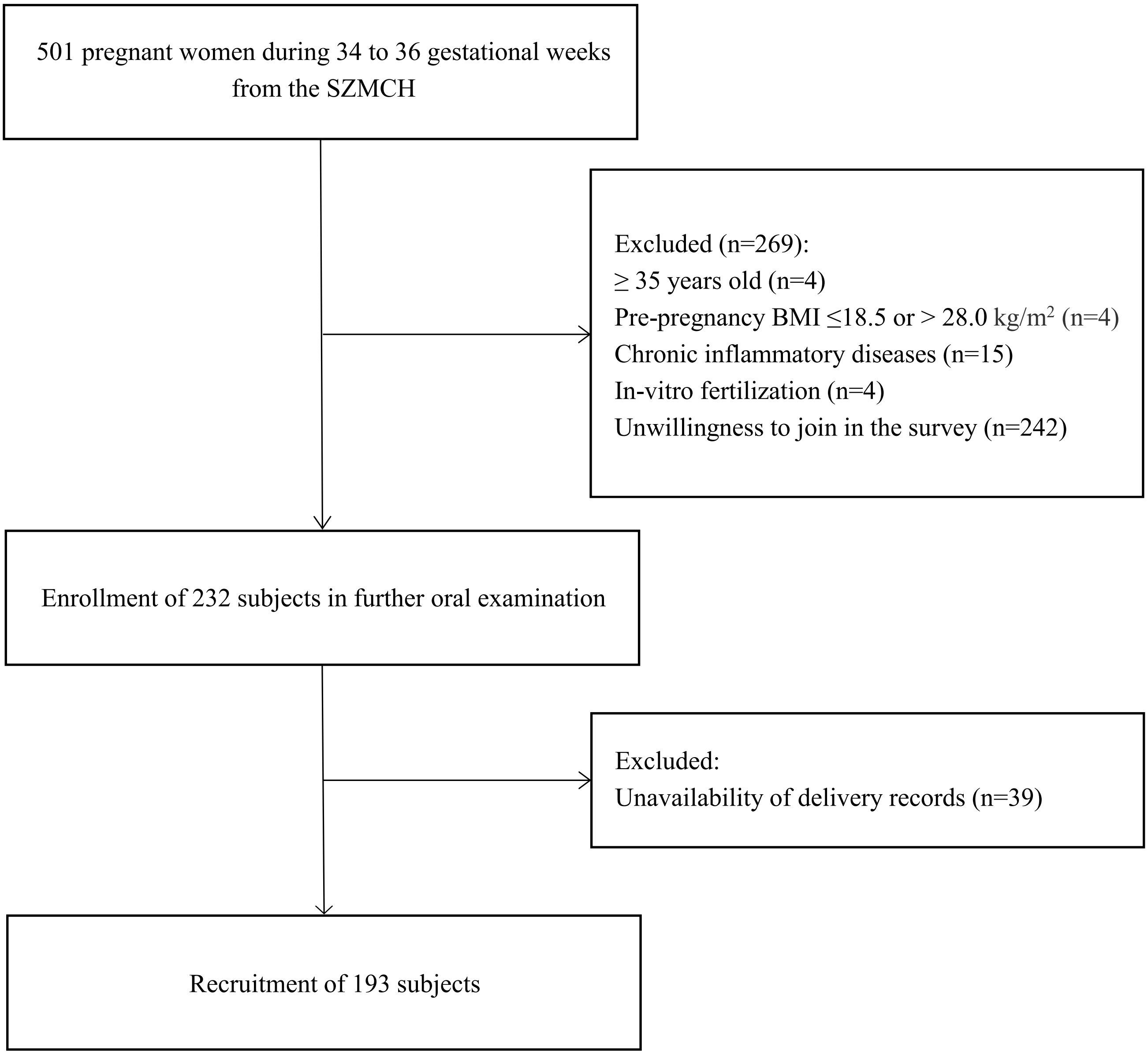

Supplementary Figure 1. The flow chart of subject recruitment. SZMCH: Shenzhen Maternity & Child Healthcare Hospital; BMI: body mass index.

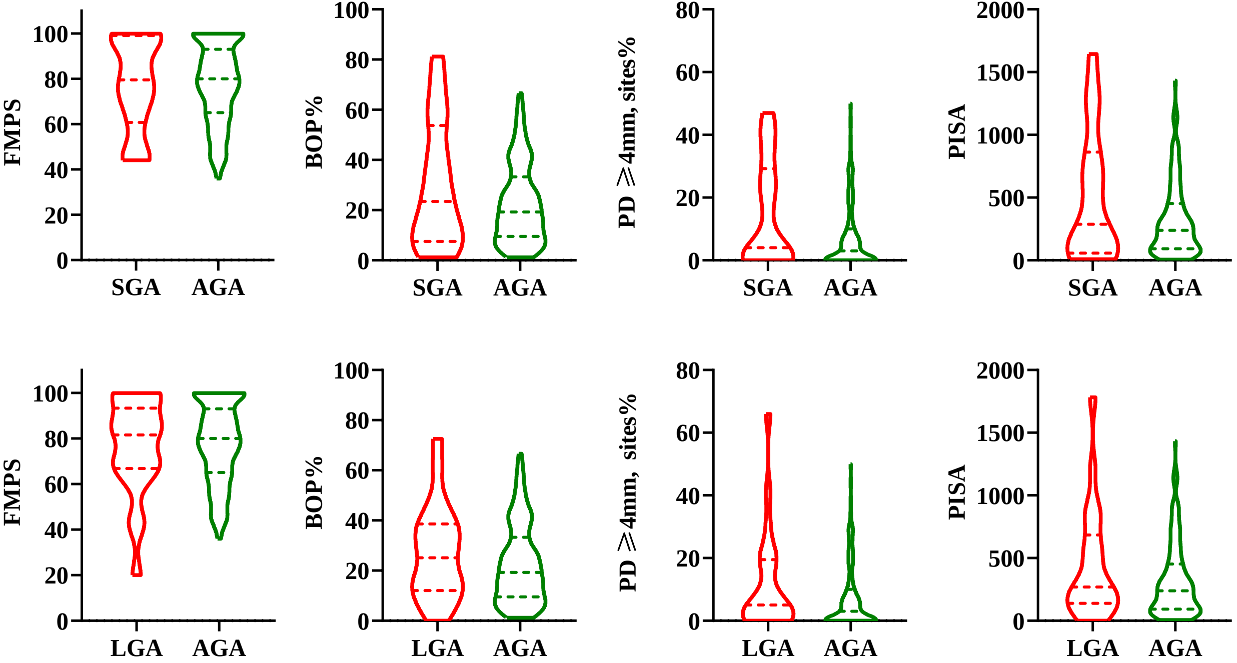

Supplementary Figure 2. Periodontal parameters in both SGA and LGA groups with reference to the AGA group. FMPS: full-mouth plaque score; BOP: bleeding on probing; PD: probing depth; PISA: the periodontal inflammed surface area.
